# Physical exercise in amyotrophic lateral sclerosis: a potential co-adjuvant therapeutic option to counteract disease progression

**DOI:** 10.3389/fcell.2024.1421566

**Published:** 2024-08-02

**Authors:** Gianmarco Fenili, Silvia Scaricamazza, Alberto Ferri, Cristiana Valle, Maria Paola Paronetto

**Affiliations:** ^1^ Department of Movement, Human and Health Sciences, University of Rome “Foro Italico”, Rome, Italy; ^2^ Laboratory of Molecular and Cellular Neurobiology, Fondazione Santa Lucia IRCCS, Rome, Italy; ^3^ Laboratory of Metabolomics, Fondazione Santa Lucia IRCCS, Rome, Italy; ^4^ Institute of Translational Pharmacology (IFT), Consiglio Nazionale Delle Ricerche (CNR), Rome, Italy

**Keywords:** ALS, physical activity, muscle atrophy, neurodegenerative disease, exercise

## Abstract

Amyotrophic lateral sclerosis (ALS) is a fatal disorder characterized by the selective degeneration of upper and lower motor neurons, leading to progressive muscle weakness and atrophy. The mean survival time is two to five years. Although the hunt for drugs has greatly advanced over the past decade, no cure is available for ALS yet. The role of intense physical activity in the etiology of ALS has been debated for several decades without reaching a clear conclusion. The benefits of organized physical activity on fitness and mental health have been widely described. Indeed, by acting on specific mechanisms, physical activity can influence the physiology of several chronic conditions. It was shown to improve skeletal muscle metabolism and regeneration, neurogenesis, mitochondrial biogenesis, and antioxidant defense. Interestingly, all these pathways are involved in ALS pathology. This review will provide a broad overview of the effect of different exercise protocols on the onset and progression of ALS, both in humans and in animal models. Furthermore, we will discuss challenges and opportunities to exploit physiological responses of imposed exercise training for therapeutic purposes.

## Graphical Abstract

**Figure:**
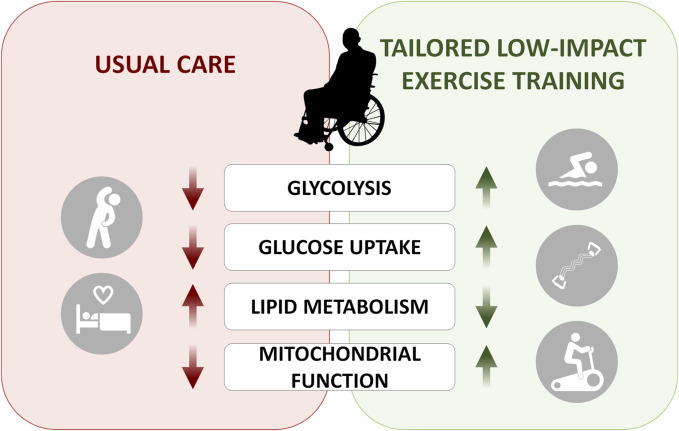
Energy metabolism is dysregulated in ALS patients. On the left panel, metabolic processes shown to be affected in ALS patients can be partially rescued with tailored low-impact exercise training protocols.

## 1 Introduction

Amyotrophic lateral sclerosis (ALS) is a fatal disorder characterized by the selective degeneration of upper motor neurons in the primary motor cortex and lower motor neurons in the brainstem and spinal cord, leading to progressive muscle weakness and atrophy (Masrori and Van Damme, 2020). Overall survival varies from a few months to decades, but on average death occurs between 2.5 and 5 years from the diagnosis (Cozzolino et al., 2008), usually for respiratory failure (Masrori and Van Damme, 2020).

ALS can be classified in two different forms depending on the region of the primary degeneration: bulbar or spinal. The spinal form (two-thirds of the cases) affects the muscles of the limbs and the trunk, and causes muscular weakness and atrophy, cramps and fasciculations. The bulbar form (one-third of the cases) affects at first the muscle of the lips, tongue and throat, and results in dysarthria and dysphagia (Hulisz, 2018). Approximately, 90% of ALS cases are sporadic (sALS), while 10% are familial (fALS). Particularly, pathogenic variants in superoxide dismutase 1 (SOD1), TAR DNA‐binding protein (*TARDBP*), fused in sarcoma (*FUS*) and chromosome 9 open reading frame 72 (*C9orf72*) genes, account for approximately 60% of the familial cases and about 10% of sporadic ALS (Akçimen et al., 2023). SOD1 is an intracellular antioxidant enzyme which protects cells by regulating basal levels of reactive oxygen species (ROS) arising from mitochondrial and cytosolic superoxide ions (Trist et al., 2021). To date, over 140 distinct *SOD1* mutations have been identified, but the precise mechanisms underlying the effect of *SOD1* mutants on mitochondrial metabolism remain unclear. Nevertheless, ALS is considered a multifactorial disease, due to a combination of risk-genotypes that interact with environmental factors, impacting and accelerating the neurodegenerative cascade (Al-Chalabi and Hardiman, 2013).

Although numerous preclinical and clinical trials have been performed, a cure for ALS does not exist yet. Indeed, since 1941, more than 60 compounds, with different mechanisms of action, have been evaluated in clinical trials for ALS treatment (Petrov et al., 2017), but only four of them have been approved by FDA for clinical use: riluzole (Rilutek, Tiglutik, Exservan), edaravone (Radicava), tofersen (Qalsody), and AMX0035 (Relyvrio), which was removed from the market in april 2024 due to negative topline data (NCT03127514). The “usual care” for ALS patients consists of treatments that slow the progression of the disease: to date, riluzole is the only treatment that has been shown to prolong survival in ALS (Bensimon et al., 1994), offered to all patients as early as possible (Miller and Appel, 2017).

Several studies documented higher incidence and lower age onset of ALS in high profile athletes, leading to the hypothesis that strenuous, repetitive exercise may represent an environmental risk factor to develop the disease (Chio et al., 2009). To date, the role of regular exercise and fitness in the pathogenesis and treatment of ALS is still controversial. ALS is commonly known as Lou Gehrig’s disease, from a famous professional baseball player who was afflicted by this devastating neurodegenerative disorder during the late 1930s. Several studies have suggested that people with active lifestyle and reduced body fat have increased risk to develop ALS (Scarmeas et al., 2002), supporting the hypothesis that heavy exercise could represent a suspected risk factor for ALS. Consistently, frequent, and strenuous physical activity seems to increase the penetrance of ALS, particularly those patients with a predisposing genotypic background as *C9ORF72* expansion have a higher risk to develop exercise-aggravated disease. (Julian et al., 2021).

The role of intense physical activity in the etiology of ALS has been debated for several decades (Harwood et al., 2009), without reaching a clear conclusion. Most athletes and physically active individuals do not develop ALS. Instead, regular physical activity has been associated with increased quality of life and has shown neuroprotective properties, ameliorating neurological impairment in different neurodegenerative processes, even hindering age-related neuronal loss. Physical activity can also enhance neurogenesis, implementing neuronal plasticity (Radak et al., 2016).

A deeper understanding of the potential interaction between genetic and environmental factors would be instrumental to deepen this debate, and for the development of preventive strategies for patients and their family members. Recent reviews have tried to answer this question, to understand whether physical activity could be considered a factor in the etiology of ALS (Chapman et al., 2023). On the other hand, considering that physical activity is able to induce cellular adaptations in the brain, spinal cord, and skeletal muscles that could counteract the oxidative stress complication of ALS, it is conceivable that exercise could be beneficial for ALS patients (Elbasiouny and Schuster, 2011; Kincaid and Bossy-Wetzel, 2013).

Physical activity represents one of the most commonly prescribed therapies, either in terms of prevention or as non-pharmacological adjunctive treatment for several chronic conditions (Vina et al., 2012). Regular moderate-intensity training reduces oxidative stress (Radak et al., 2008a; Radak et al., 2008b), decreases levels of inflammatory markers in elderly (Sellami et al., 2021), helps to preserve cardiovascular fitness and brain function (Hotta et al., 2017), and protects individuals from the negative effects of stress on cell aging (Rebelo-Marques et al., 2018). In skeletal muscle, it attenuates mitochondrial deficits, thus improving muscle function (Wyckelsma et al., 2017). However, strenuous exercise training generates high levels of reactive oxygen species (ROS) known to cause oxidative stress and activate pathogenic pathways, thus accelerating the aging process (Sahl et al., 2017). In ALS, mitochondrial dysfunction and oxidative stress are tightly dependent on each other and represent the basis of the redox dysregulation, which contributes, at least in part, to death of motor neurons. Mitochondria are the main site of production of ROS; hence, impairment of mitochondrial function, as in ALS, increases the oxidative stress (Carrì et al., 2015). However, exercise ROS production could also represent a potentially harmful stressor able to promote adaptive changes, enabling to tolerate subsequent stress. According to the mitohormesis, in response to ROS perturbation the mitochondria can initiate and transduce a signal transduction pathway coordinating a transcriptional response which results in both mitochondrial and non-mitochondrial adaptations, and maintains cellular homeostasis (Merry and Ristow, 2016).

An additional layer of complexity is given by the impact of ALS on energy metabolism. Indeed, altered metabolic homeostasis represents an early event in ALS, documented both in patients and in mouse models of ALS, with weight loss and reduced fat mass, altered glucose and lipid handling, and increased resting energy expenditure (Dupuis et al., 2004; Dupuis et al., 2011; Scaricamazza et al., 2020; Steyn et al., 2020). Notably, these metabolic alterations also affect the neurodegenerative process: as a result, increased dietary lipid content offers neuroprotection and extends survival in mouse models of ALS (Dupuis et al., 2004; Dupuis et al., 2011), whereas restricting calorie intake exacerbates motor symptoms (Pedersen and Mattson, 1999; Fergani et al., 2007). Affected skeletal muscles decrease using glucose as a source of energy but use lipids instead and this chronic pathologic alteration is exacerbated with disease progression (Steyn et al., 2020). The switch toward lipid use in glycolytic muscle precedes neuromuscular junction denervation in mouse models (Dupuis et al., 2009; Palamiuc et al., 2015). Remarkably, administration of dichloroacetate (DCA), a halogenated organic acid that inhibits the activity of PDK and facilitates the entry of pyruvate into the Krebs cycle and the oxidation of glucose, is sufficient to force metabolism toward glucose oxidation, reverting metabolic imbalance (Palamiuc et al., 2015).

Given these reported findings, specific physical exercises are expected to differentially shift the muscular energy metabolism either toward an oxidative pattern, lipidic, in case of low-intensity exercise, or toward a glycolytic metabolism, in case of high-intensity exercise (Romijn et al., 1993; van Loon et al., 2001).

Herein, we review and discuss the effect of exercise training on the onset and progression of ALS, both in humans and in animal models, with particular emphasis on novel therapeutic options intended to take advantage of the physiological response to exercise, suggesting the possible set up of personalized therapies.

## 2 Physical activity and exercise training in ALS

“Physical activity” and “exercise” describe different concepts, often confused with one another. Physical activity is defined as any body movement produced by skeletal muscles that require energy and can be categorized into occupational, sports, conditioning, household, or other activities. “Exercise” represents a subset of physical activity that is planned, structured, and repetitive and has, as final or intermediate objective, the improvement or maintenance of physical fitness, physical performance, or health (Caspersen et al., 1985).

The molecular basis of responses to acute and chronic exercise training have been extensively studied both in humans and in mice affected by ALS. While some researchers have found that exercise may improve the quality of life of patients, other studies have shown that exercise may paradoxically impair their neuromuscular function (Angelini and Siciliano, 2021). For sure, exercise training can offer physiological and psychological benefits for patients with ALS, particularly during the early stages of the disease (Lunetta et al., 2016). One important effect of physical activity, besides its positive impact on mental and behavior status, is that exercise improves metabolism in skeletal muscle by enhancing both glucose metabolism and mitochondrial biogenesis, which, in turn, strengthens the antioxidative defense.

So, what is the main difference between the exercise protocols administrated?

Resistance exercise entails repetition of dynamic muscle-shortening (concentric) and muscle-lengthening (eccentric) contractions against external load (performed with the help of weight machines or resistance bands). It improves muscle strength and force, helps maintaining skeletal muscle function, minimizes the risk of disability, also favoring muscle hypertrophy (Smith et al., 2023).

Endurance exercise (or aerobic exercise) is associated with training-induced improvements in maximal oxygen consumption (VO_2MAX_). This exercise type is divided according to intensity: low (<50%), moderate (∼50%–79%) or high intensities (≥80%) of VO_2MAX_ (Smith et al., 2023).

## 3 Exercise training protocols in mouse models of ALS

To elucidate whether physical exercise displays a positive or negative effect on ALS onset and progression, different training protocols have been administrated in animal models of ALS, summarized in Table 1. Particularly, transgenic mice that express G93A mutant *SOD1* (*SOD1* ^*G93A*^) human gene have been generated (Ripps et al., 1995) and extensively used for this purpose. *SOD1* ^*G93A*^ mice develop progressive lower motor neuron weakness and increased oxidative stress and reproduce the clinical and pathological hallmarks of ALS (Ripps et al., 1995). Furthermore, the *Sod1* ^*G86R*^ mouse models were developed, which express the missense mutation Gly86 to Arg of the murine SOD1 enzyme (Ripps et al., 1995), reported in the corresponding amino acid residue (position 85) of some fALS patients (Deng et al., 1993; Rosen et al., 1993).

**TABLE 1:** Training protocols in mouse models of ALS. Specific protocols, outcomes and Reference of the studies are indicated.

Training protocol	Outcomes	Ref. Study
*Running-based training protocol in ALS mice (Treadmill)*		
Treadmill running at 17 m/min for 30 min, 5 days/week	Increased survival in males and females	Kirkinezos et al. (2003)
Treadmill running at 16 m/min for 45 min, 5 days/week	Delayed onset of disease in female Decreased survival in males	Veldink et al. (2003)
Treadmill running at 20 m/min for 45 min, 5 days/week +/- nandrolone	No changes on the onset of disease Increased motor neuron counts after PA	Kassa et al. (2017)
Moderate intensity: treadmill running at 10 m/min, for 30 min, 3 days/week	Delayed onset of Motor neuron deficit	Carreras et al. (2010)
High intensity: treadmill running at 20 m/min, for 60 min, 5 days/week	Hastened onset Motor neuron density Decreased survival	Carreras et al. (2010)
Running in a speed-regulated treadmill (max. 13 m/min), for 30 min, 5 days/week	No changes in the Onset of disease No changes in Survival	Deforges et al. (2009)
Running in a speed-regulated treadmill (from 10 m/min to max. 22 m/min), increasing from 15 to 60 min, 5 days/week	Anticipated onset Decreased survival	Scaricamazza et al. (2024)
*Running-based training protocol in ALS mice (wheels)*		
2 h daily exposure to running wheels	Increased Survival	Kaspar et al. (2005)
6 h daily exposure to running wheels	Increased Motor performance Increased Survival	Kaspar et al. (2005)
12 h daily exposure to running wheels	Increased Motor performance Increased Survival	Kaspar et al. (2005)
10 h daily exposure to running wheels: 40 × 10 min of running, 5 min rest	No changes in the Onset of disease No changes in Motor performance Increased Survival	Liebetanz et al. (2004)
multiple exercise sessions at asymptomatic and pre-symptomatic stages in an automated home-cage running-wheel system for 3 months	Anticipated onset Decreased survival	Golini et al. (2023)
*Swimming-based training protocol in ALS mice*		
Swimming (max. 5 L/min), for 30 min, 5 days/week	Delayed Onset of disease Increased Survival Increased Motor performance	Deforges et al. (2009)
Swimming (max. 5 L/min), for 30 min, 5 days/week	Increased Glucose tolerance Increased Plasma lactate Increased GLUT4 expression Increased Lipid synthesis	Desseille et al. (2017)
Swimming (max. 5 L/min), for 30 min, 5 days/week	Increased Motor performance Decreased Mitochondrial dysfunction Increased malate synthase activity	Flis et al. (2019)
Swimming (max. 5 L/min), for 30 min, 5 days/week	Increased GPx activity	Dzik et al. (2021)

In mice, three commonly used protocols for exercise training have been developed: swimming, voluntary wheel running, and “forced” wheel or treadmill running. Treadmill running and wheel running induce adaptations in mice associated with endurance exercise training (Kemi et al., 1985; Waters et al., 2004; Massett and Berk, 2005). However, while with the treadmill running protocol the total amount of work performed can be precisely established through the selection of exercise testing and training parameters, the voluntary wheel running does not offer this advantage. Unlike for humans, there are no well-accepted standards for exercise training paradigms or levels of activity required for optimal changes in exercise capacity or other training adaptations (Fuller and Thyfault, 1985). Unlike treadmill exercise, voluntary wheel-running enables mice to run freely and at a lower intensity. Although this practice may cause individual differences among mice in the amount of exercise they get, the mice do not experience much stress.

Swimming-based training protocol display changes in skeletal muscle energetic metabolism of *SOD1* ^*G93A*^ mice, shifting energetic fuels to the anaerobic glycolytic pathway, as detailed below.

Notably, mouse strain, sex, and age have been reported to influence exercise training responses. For example, male mice had significantly greater biochemical adaptations to exercise training than female mice (Massett and Berk, 2005). Moreover, the adaptation to exercise training strictly depends on factors such as training load, duration, and frequency.

The first studies investigating the role of physical exercise on ALS onset and progression demonstrated a beneficial effect achieved by an endurance training protocol in *SOD1* ^*G93A*^ mice (Kirkinezos et al., 2003). In particular, Kirkinezos and colleagues performed a treadmill protocol with a 5 days per week exercise regimen of 30 min at 17 m/min, showing a significant increase in the life span of both male and female *SOD1* ^*G93A*^ mice (Kirkinezos et al., 2003). Conversely, Veldink and colleagues found a positive neuroprotective effect of their treadmill protocol only for female *SOD1* ^*G93A*^ mice. In detail, the endurance exercise training, consisting of 45 min per day, 5 days per week at 16 m/min, was able to delay the onset of the disease in female but not in male *SOD1* ^*G93A*^ mice, that showed instead a hastened death (Veldink et al., 2003). In either case, the authors highlighted the potential role of sex hormones as a possible explanation for their gender-specific response to exercise. A similar protocol showed no impact on disease onset, whereas a beneficial effect on survival of motoneurons was demonstrated (Kassa et al., 2017). In this latter study the use of anabolic steroids, still debated in epidemiological studies on patients and murine models of ALS, was also assessed. The authors showed that nandrolone treatment markedly enhanced motoneuron loss; its detrimental effect was reverted by the combination with exercise, suggesting a potential neuroprotective effect of physical exercise (Kassa et al., 2017).

Carreras and colleagues found that moderate exercise (30 min of exercise per day, 3 days a week at 10 m/min) was able to delay the onset of motor deficit by over a week in *SOD1* ^*G93A*^ mice, whereas high intensity exercise (60 min of exercise per day, 5 days per week at 20 m/min) slightly but significantly hastened the onset of motor performance deficits (Carreras et al., 2010). More recently, Scaricamazza and colleagues demonstrated that intense endurance exercise exerted a detrimental effect on *SOD1* ^*G93A*^ mice (Scaricamazza et al., 2024). Particularly, starting the training far from the onset, they demonstrated that intense endurance exercise was able to bring the onset of the disease forward and to worsens the progression of symptoms by hastening the motor-skill impairment and accelerating the denervation process and the motor neuron death. These data suggest that intense endurance exercise could represent a risk factor in ALS (Scaricamazza et al., 2024).

Running wheels protocols provided more positive results than treadmill protocols, as mentioned above. Mice with the opportunity to exercise with wheels voluntarily choose to do so; even those with debilitating conditions, such as symptomatic *SOD1* ^*G93A*^ mice, exhibit a strong motivation to use an exercise wheel. Hence, different enrichment strategies, including running wheels, affect disease progression and may have implications for experimental outcomes (Sorrells et al., 2009). A short 2-h exposure to the running wheels showed a significant 7-day extension in median survival compared with non-running animals (Kaspar et al., 2005), whereas 6–12-h exposure to the running wheels provided significant benefits to motor functions (Kaspar et al., 2005). Even vigorous training protocols, as achieved by chronic exposure to motor-driven running wheels, did not negatively impact disease onset in *SOD1* ^*G93A*^ mice, but increased survival of 1 week (Liebetanz et al., 2004). Interestingly, exercise training was shown to exert a remarkable synergistic effect with insulin-like growth factor-1 administration, promoting motor neuron survival, attenuating astrogliosis, improving motor function, and extending survival (Kaspar et al., 2005).

Notably, the administration of multiple exercise sessions at an early pre-symptomatic disease stage through a running wheels system to *SOD1* ^*G93A*^ mice expressing low copy of mutant *SOD1*, significantly worsened disease course predating the symptoms onset (Golini et al., 2023). This latter evidence allows to hypothesize a negative impact of intense physical exercise, if administered at a very early age.

By comparing running-to swimming-based exercise protocols, Deforges and colleagues demonstrated that, in *SOD1* ^*G93A*^ mice, a swimming-based training protocol was able to sustain the motor function limiting astrogliosis and hypertrophic processes, with a remarkable increase in the life span by about 25 days (Deforges et al., 2009). The magnitude of this beneficial effect is one of the highest among those induced by any therapeutic strategy in ALS. Unlike running, swimming significantly delayed spinal motoneuron death and, more specifically, the motoneurons of large soma area. Analysis of the muscular phenotype revealed a swimming-induced relative maintenance of the fast phenotype in fast-twitch muscles (Desseille et al., 2017). Moreover, high intensity swimming exercise significantly improved glucose metabolism, which is strongly impaired in *SOD1* ^*G93A*^ mice, as well as in ALS patients. These swimming-induced benefits were associated with changes in skeletal muscle energetic metabolism, leading to energetic fuel shifts toward glucose re-use and fat deposition. In particular, the increase in GLUT4 expression induced by swimming was instrumental to switch energetic fuel, feeding the glycolytic pathway.

Thus, if running-based trainings showed to reinforce the oxidative pathway contributing to the neurodegenerative process, swimming-based training worked as modulator of skeletal muscle energy metabolism, with concomitant improvement of skeletal muscle function. Notably, swim training significantly decreased the reduction in muscle strength clearly visible at the symptomatic stage of ALS (Flis et al., 2018). Swim-training is characterized by non-weight bearing exercises that minimize damage to muscle fibers, reducing oxidative stress, and improving muscle energy metabolism at the terminal stage of the disease (Flis et al., 2018). As discussed above, energy metabolism dysfunction is a characteristic sign of ALS disease and defects in skeletal muscle energy metabolism deeply contribute to disease progression (Dupuis et al., 2011). However, Flis and colleagues report that after swim training the electron transport chain did not change between wild type and *SOD1* ^*G93A*^ mice, in contrast significant changes were observed in citrate synthase, malate dehydrogenase, and cytochrome c oxidase (Flis et al., 2019). In ALS skeletal muscles, the increase in the activity of malate dehydrogenase and cytochrome C is accompanied by a decrease in citrate synthase activity, activating a compensatory mechanism to maintain the production of oxalacetate in the muscles, and thus the Krebs cycle. Administration of the swimming protocol was able to significantly increase the citrate synthase activity while reducing the malate dehydrogenase activity, thus maintaining ATP production capacity by mitochondria (Flis et al., 2019). In this way, swimming-training resulted neuroprotective and delayed muscle wasting, as demonstrated by the grip strength test (Flis et al., 2019). Moreover, the activation of the BDNF/TrkB neurotrophic signaling, which retrogradely modulate neurotransmission and protect neuromuscular junctions and motoneurons (Just-Borràs et al., 2020), together with the increased glutathione peroxidase activity (Dzik et al., 2021), could contribute, at least in part, to the beneficial effect of the swimming training protocol.

## 4 Exercise training protocols in ALS patients

In patients with ALS, various training protocols have been proposed to evaluate their potential beneficial effects. These protocols are summarized in Table 2. However, it is noteworthy that these studies are constrained by the significant intrinsic heterogeneity among ALS patients. Furthermore, it is important to acknowledge that epidemiological studies conducted in this patient population frequently rely on retrospective investigations, which may introduce biases and potentially result in an overestimation or underestimation of activity levels.

**TABLE 2:** Training protocols performed in ALS patients. Specific protocols, outcomes and reference of the studies are indicated.

Training protocol	Outcomes	Ref. Study
*Resistance training*		
Resistance exercises to upper extremities (2 sets × 10 reps, 5 min rest), 6 days/week, for 75 days	Beneficial effect	Bohannon (1983)
Resistance training and stretching for the trunk muscles, upper and lower limbs, 6 days/week, for 6 months	Beneficial effect; higher respiratory function	Kitano et al. (2018)
Shoulder, elbow, hip, knee flexion; elbow, knee extension; grip; increase intensity, 3 days/week for 6 months	Beneficial effect	Clawson et al. (2018)
Moderate-load and moderate-intensity resistance exercise program to upper and lower extremities, 3 days/week, for 6 months	Beneficial effect	Bello-Haas et al. (2007)
Active exercises against gravity in six muscle groups in the upper and lower limbs (3 sets × 3 reps each muscle group) performed daily for 2 weeks each month, for 6 months	No significative effect	Lunetta et al. (2016)
Passive exercises consisting of 20 min of 20 flexion–extension movements per minute in the upper and lower limbs, daily for 2 weeks/month, for 6 months	No significative effect	Lunetta et al. (2016)
Passive, active and cycle ergometer exercises, strictly supervised; 2/week, for 6 months	No effect on survival, reduced motor deterioration	Lunetta et al. (2016)
Resistance exercises targeting both upper and lower body (2 sets × 5 reps at 6RM), 2–3 days/week, for 12 weeks	Negative effect	Jensen et al. (2017)
*Endurance training*		
Endurance exercises to whole body, against modest loads, lasted 15 min twice daily, for 12 months	Beneficial effects	Drory et al. (2001)
Walking on a weight-supported treadmill for 30 min (6 sets × 5 min, 5 min rest), 3 days/week, for 8 weeks	Beneficial effect	Sanjak et al. (2010)
10 min of upper limb exercise followed by 10 min lower limb exercise using a minicycle, 40%–70% of target HR or 13–15 in Borg scale, 3 days/week for 6 months	Beneficial effect	Clawson et al. (2018)
Aerobic exercise therapy in cycle ergometer; 50 min, 3 sessions a week, for 16 weeks	No significant effect	van Groenestijn et al. (2019)
Reclining stepped aerobic exercise of moderate intensity. 70 steps/minute; 40 min, 3 sessions a week, for 4 weeks	No significant effect	Sivaramakrishnan and Madhavan (2019)
Ramp treadmill protocol. (exercise performed with the assistance of the ventilator Bipap STD^®^), for 12 months	Beneficial effects	Pinto et al. (1999)
Moderate aerobic exercise on a treadmill with non-invasive ventilation and body weight supporting system, 2 days/week, for 6 months	Beneficial effect	Braga et al. (2018)
*Combined endurance and resistance training*		
Moderate/high intensity strength and endurance exercises; 30 min, 7 sessions a week, for 2 weeks	No significative differences	Kato et al. (2018)
Submaximal aerobic exercise 65% HR and 80% strength RM; 50 min, 7 sessions a week, for 5 weeks	Beneficial effect	Merico et al. (2018)
Moderate/high intensity aerobic and strength exercise; 50 min, 3 sessions a week, for 12 weeks	Beneficial effect	Ferri et al. (2019)
High frequency aerobic and resistance training (5/week) vs aerobic exercise, low frequency (2/week); 45 min, for 10 weeks	No significative differences	Zucchi et al. (2019)

The first exercise protocol on ALS patients was performed by Bohannon and colleagues (Bohannon, 1983); they reported an increase in static strength in the muscles of upper extremities, suggesting a beneficial effect of resistance training. These results were confirmed by Kitano (Kitano et al., 2018), showing that resistance and stretching exercise are safe and feasible for patients at early stage of ALS, especially in respiratory function. In 2017, Clawson and colleagues (Clawson et al., 2018) demonstrated that resistance training ameliorated patient function more than standard care (Bello-Haas et al., 2007). By comparing individuals who received ‘standard care’ with a group who underwent a strictly monitored exercise program (SMEP), it was shown that the SMEP group obtained beneficial effects from the training protocol (Lunetta et al., 2016). In particular, the SMEP group was further divided into three subgroups: one performing an active exercise program plus cycloergometer activity, a second subgroup performing only active exercise, and a third subgroup performing passive exercises. At a single 180-day endpoint of the study, a difference in the ALSFRS-R was observed between those who underwent the SMEP and those who received ‘standard care’, but not at earlier time-points. Although no effect on survival was demonstrated, the obtained results suggest that a strictly monitored exercise program may significantly reduce motor deterioration in ALS patients (Lunetta et al., 2016). The only study with negative results reported that did not attenuate disease progression with possible negative effects on skeletal muscle (observed by functionality, voluntary muscle activation and cross-sectional area), with loss of muscle strength and power (Jensen et al., 2017).

Collectively these studies indicate that resistance training increases muscle strength, power, and force. Although these parameters are fundamental to ameliorate the lifestyle, they do not reduce the disease progression.

In 2001, Drory and colleagues performed a comparison between a daily endurance exercise program and usual daily care (Drory et al., 2001). They noticed that a tailorized, moderate range of motion training displays a beneficial effect on muscle endurance and a mild, temporary positive effect on the motor deficit, disability, fatigue, and health-related quality of life (Sanjak et al., 2010). Endurance training was also shown to display an improvement in work capacity and gait function in ALS patients dependent on walking aids devices (Sanjak et al., 2010). The comparison of endurance exercise to SROM (Stretching/Range of Motion) revealed that this latter appeared safe and well tolerated, whereas endurance training appeared too vigorous and had lower overall compliance (Clawson et al., 2018). Van Groenestijn and colleagues (van Groenestijn et al., 2019) proposed for the first-time aerobic exercise therapy (AET). Due to the small number of patients who completed the exercise protocol, they concluded that AET should not be included in usual care therapy. As specified in the United Kingdom clinical guidelines for motor neuron disease, the “usual care” comprises medications and treatments such as non-invasive ventilation, physiotherapy and gastrostomy, and access to other hospital-based and community-based services, including equipment and adaptations, orthotics, respiratory, gastroenterology, clinical psychology, neuropsychology, and counselling, as well as social care services. Even if a stepping exercise was well tolerated by all study participants, no significant improvements in clinical parameters were documented (Sivaramakrishnan and Madhavan, 2019).

In general, the beneficial effects of endurance exercise appear enhanced using non-invasive ventilation.

To maximize the beneficial effects of exercise therapy, a few clinical trials have included a combination of aerobic and resistance training protocols. However, they did not show differences in comparison to usual care (Kato et al., 2018; Merico et al., 2018; Ferri et al., 2019; Zucchi et al., 2019).

## 5 Exercise impact on skeletal muscle metabolism

Physical exercise impacts on specific energy fuels depending on the intensity and duration. An acute bout of exercise leads to the activation of signaling pathways driving short-term and long -term systemic adaptations. As first, breakdown of ATP and phosphocreatine, provides a substantial quantity of high-energy phosphate in a remarkably short time, typically within milliseconds. If the physical activity last up to 1 min, also anaerobic glycolysis is used for energy production, giving rise to lactate by lactate-dehydrogenase (LDH). Glycolysis rate-limiting enzyme phosphofructokinase (PFK) activation is promoted by the increase of glucose cytoplasmatic concentration. Eventually, for exercise longer than 1 min, oxidative phosphorylation is the major ATP-generating pathway. Increased metabolic demands are accomplished through mechanisms that are mostly mediated by the sympathetic nervous system, such as lipolysis, mobilization of hepatic glycogen stores, that favor the increase in available glucose and free fatty acids for the exercising skeletal muscles (Hawley et al., 2014). Chronic aerobic exercise upregulates oxidative metabolism in a time-dependent manner, immediately reverted to baseline following abstinence from exercise. Indeed, skeletal muscles undergo multiple adaptive mechanisms, including increased mitochondrial biogenesis, expression of fatty acid transporters, activity of oxidative enzymes and of those involved in the electron transport chain in the mitochondria, contributing to skeletal muscle hypertrophy (Hawley et al., 2014).

Changes in mitochondrial bioenergetics might underlie defects in exercise capacity of ALS muscles. As anticipated above, a switch from glucose to lipid metabolism occurs early in the disease process and prior to any detectable motor and clinical symptoms in animal models (Scaricamazza et al., 2020). Thus, the decreased capacity to tolerate acute physical exercise that solicits anaerobic metabolism in muscle occurs before muscle weakness or denervation (Palamiuc et al., 2015; Scaricamazza et al., 2020). This low resistance to intense exercise seems in contrast with the enhanced endurance capacity observed during acute aerobic exercise. This observation suggests that ALS mice acquire new properties in muscle fibers that enhance global aerobic capacity and promote endurance ability (Pradat et al., 2010; Palamiuc et al., 2015).

**FIGURE 1:**
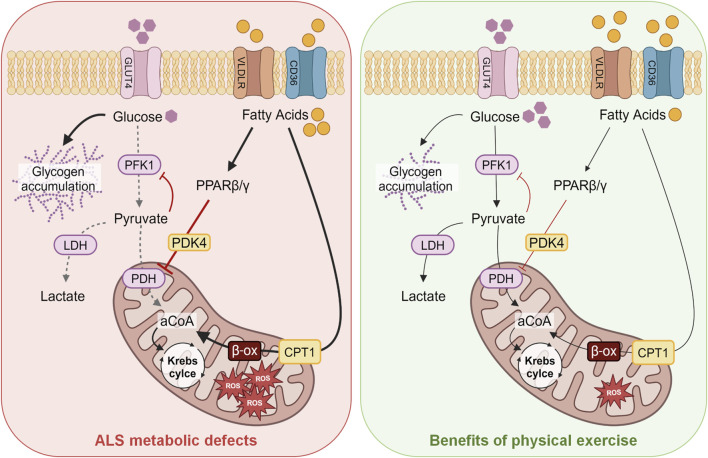
Schematic model of the energetic metabolism of ALS skeletal muscles and their changes induced by swimming-training. The main alterations in ALS skeletal muscles observed in the glycolytic and lipidic metabolism are shown on the left (red panel), whereas the improvements in energetic fuels induced by exercise protocols, particularly swimming, are schematized on the right (green panel). Abbreviations: GLUT4, glucose transporter 4; PFK1, phosphofructokinase 1; LDH, lactate dehydrogenase; PDH, pyruvate dehydrogenase; VLDLR, very-low-density lipoprotein receptor; PPAR β/γ, peroxisome proliferator-activated receptor; PDK4, pyruvate dehydrogenase lipoamide kinase isozyme 4; CPT1, carnitine palmitoyltransferase 1; aCoA, acetyl coenzyme A; ROS, reactive oxygen species; ALS, amyotrophic lateral sclerosis.

Endurance exercise is supported by slow-twitch oxidative type I fibers, while intense exercise is supported by fast-twitch glycolytic type IIb fibers (Bassel-Duby and Olson, 2006). Remarkably, a switch in fiber type, from glycolytic to oxidative, has been described in ALS patients (Telerman-Toppet and Coërs, 1978) as well as and in mice (Deforges et al., 2009), thus explaining the different exercise capacity. The increase in endurance capacity in ALS mice might underlie a profound alteration of fuel preference in muscle fibers, paralleling altered glucose metabolism. The rate-limiting enzyme of the glycolysis is represented by the phosphofructokinase-1 (PFK1), whose inhibition leads to an increase in glycogen synthase activity and glycogen accumulation in skeletal muscle, which is in fact a characteristic of a muscle subjected to endurance training (Vestergaard, 1999), but also a characteristic of ALS mice (Palamiuc et al., 2015) (Figure 1, left panel). At symptomatic stages of disease in ALS mouse models, a fiber type switching toward more oxidative metabolism (Deforges et al., 2009) is paralleled by the increased expression of genes encoding enzymes involved in lipid metabolism (Dupuis et al., 2004; Fergani et al., 2007), which may support lipid mobilization and uptake. This can lead to increased β-oxidation by-products, thus activating the pyruvate dehydrogenase lipoamide kinase isozyme 4 (PDK4) and inhibiting pyruvate dehydrogenase (PDH) activity (Denton et al., 1975). In line with this observation, PDK4 is strongly induced in ALS mouse models and patients (Palamiuc et al., 2015). PDK4 phosphorylates PDH and inhibits the entry of pyruvate into the Krebs cycle, thus hampering glucose oxidation (Denton et al., 1975). Increased β-oxidation of fatty acids leads to the generation of lipid by-products that contribute to ROS production (Aon et al., 2014). At later stages of the disease, reduced activities of both PFK1 and glycogen synthase, together with an increase in the glycogen stores and reduced levels of pyruvate, give evidence of the inhibition of the glycolytic pathway (Figure 1, left panel). In contrast, the lipid pathway is stimulated (Dupuis et al., 2004), as documented by the increased lipid clearance in ALS mice (Fergani et al., 2007) and patients (Pradat et al., 2010).

The upregulation of the muscle-specific peroxisome proliferator-activated receptor β/δ (PPARβ/δ) could explain the shift from fast-twitch to slow-twitch fibers (Wang et al., 2004). PPARβ/δ acts as a metabolic regulator in several tissues; its activation, as upon physical exercise or long-term fasting, promotes fatty acid oxidation in skeletal muscle and induces a switch toward type I muscle fibers, resembling the fiber type transition induced by endurance training (Wang et al., 2004). Activation of PPARβ/δ enhances mitochondrial capacity and fat oxidation in the skeletal muscle (Figure 1). Thus, the metabolic imbalance in muscle fibers of ALS mice and patients represents an early event; glycolytic muscle fibers become progressively unable to use glucose as an energy substrate, switching to lipid use to maintain energy supply, as reported by Steyn and colleagues that described a decreased metabolic flexibility in muscle fibers obtained from ALS patients (Steyn et al., 2020). In particular, the authors observed an increased propensity to use lipids with respect to glucose as energy source in skeletal muscle of ALS patients. The restoration of metabolic equilibrium in glycolytic muscle fibers induced by exercise, particularly swimming endurance training, could protect muscle mitochondria and hamper oxidative stress, also preventing denervation and atrophy. Remarkably, physical exercise, particularly swimming, can lead to increased glucose uptake also by upregulating GLUT4 expression, thus contributing to improve glycolysis, as revealed by the enhanced lactate production (Desseille et al., 2017). This suggests that pyruvate is used to enhance the anaerobic glycolytic pathway in ALS muscles. Therefore, a tailored physical exercise could help in restoring the correct glucose uptake by skeletal muscle, decreasing insulin or glucose resistance observed in both mouse models and patients (Dobrowolny et al., 2018; Scaricamazza et al., 2020). The use of glucose instead of fatty acids might decrease mitochondrial overwhelming with a positive impact on oxidative stress. Conversely, reinforcing the oxidative pathway by running-based training seems to contribute to altering energetic metabolism in ALS muscle and to favor the neurodegenerative process.

## 6 Concluding remarks

ALS is a complex neurodegenerative disease where several pathological mechanisms contribute to the selective death of motor neurons, including glutamate excitotoxicity, protein aggregation, oxidative stress, neuroinflammation and dysregulation of energy metabolism (Tefera et al., 2021). In particular, overproduction of ROS overwhelms the protective defense mechanism of cells, in particular, thus contributing to neurodegenerative diseases, including ALS. These events, in turn, can cause mitochondrial dysfunction and excitotoxicity. Direct consequences of the redox imbalance are lipid peroxidation, oxidation of proteins, DNA damage, and interference of ROS with signal transduction pathways. These consequences become even more harmful when associated with inherited genetic variations. Therefore, therapeutic strategies should aim at reducing free-radical formation, and at improving clearance. On the other hand, cellular adaptations to redox imbalance can get cells used to low doses of ROS, thus buffering cellular response and contributing to mitigate direct consequences of ROS interference. In fact, beyond inducing oxidative stress, ROS play a crucial role in maintaining cellular function. In the skeletal muscle, exercise-induced ROS can promote adaptive changes, including enhanced protein synthesis, activation of insulin signaling, mitochondrial biogenesis, regulation of muscle development, gene expression, and positive modulation of antioxidants (Ristow et al., 2009). Hence, repeated exposure to sublethal stress, such as during exercise training, can enhance stress resistance and ultimately increase survival rates due to the hormesis process (Merry and Ristow, 2016). To this regard, the intake of vitamins C and E is sufficient to reduce the positive effects of exercise via ROS-related induction of PPARγ, PGC1α, and PGC1β, as well as the ROS-detoxifying enzymes, indicating that the removal of ROS can reduce, or even inhibit, the health benefits of exercise (Ristow et al., 2009). These reported findings highlight the role of ROS as signaling molecules in promoting skeletal muscle health during exercise.

Several exercise protocols have been tested in ALS patients and mouse models of the disease. Besides the neuroprotective effects exerted by any physical activity, swimmimg-based protocols displayed the most positive outcomes in mice. Particularly, swimming-based protocol resulted in a remarkable increase in the lifespan and neuroprotection, associated with changes in skeletal muscle energetic metabolism, leading to energetic fuel shifts toward glucose (Deforges et al., 2009). Although swimming is suggested as an advantageous type of exercise in many neurological disorders (Ayán and Cancela, 2012), its beneficial role has not yet been experimentally validated in ALS patients.

Overall, endurance training with a supplemental support such as ventilation or weight support seems to have positive effects on respiratory capacity, functionality, and physical performance in ALS patients, but further studies investigating the therapeutic benefits of exercise with a larger number of participants are needed to confirm these findings.

Future work should also consider the interaction of exercise with the full spectrum of genetic changes linked to ALS, both in humans and in mice. To date, however, small sample sizes, non-representative control populations, heterogeneous disease-stage of patients, have strongly affected the interpretation of results. Thus, while promising, more pre-clinical and clinical studies are needed to elucidate the role of exercise training in ALS patients.

In conclusion, disturbances in energetic metabolism are clearly linked to the selective vulnerability of motor neurons, thus, a therapeutic strategy tackling energy metabolism through tailored exercise protocols in ALS patients may pave the ground for future combined therapeutic interventions.
